# The Assessment of the Safety Profile of Selective Serotonin Reuptake Inhibitors Versus Other Antidepressants: Drug–Drug Interaction Insights from EudraVigilance

**DOI:** 10.3390/jcm14041208

**Published:** 2025-02-12

**Authors:** Carmen Maximiliana Dobrea, Claudiu Morgovan, Adina Frum, Anca Butuca, Adriana Aurelia Chis, Anca Maria Arseniu, Steliana Ghibu, Razvan Constantin Vonica, Felicia Gabriela Gligor, Ioana Rada Popa Ilie, Andreea Loredana Vonica Tincu

**Affiliations:** 1 Preclinical Department, Faculty of Medicine, “Lucian Blaga” University of Sibiu, 550169 Sibiu, Romania; carmen.dobrea@ulbsibiu.ro (C.M.D.); claudiu.morgovan@ulbsibiu.ro (C.M.); adriana.chis@ulbsibiu.ro (A.A.C.); anca.arseniu@ulbsibiu.ro (A.M.A.); razvanconstantin.vonica@ulbsibiu.ro (R.C.V.); felicia.gligor@ulbsibiu.ro (F.G.G.); loredana.vonica@ulbsibiu.ro (A.L.V.T.); 2 Department of Pharmacology, Physiology and Pathophysiology, Faculty of Pharmacy, “Iuliu Haţieganu” University of Medicine and Pharmacy, 400012 Cluj-Napoca, Romania; steliana.ghibu@umfcluj.ro; 3 Department of Endocrinology, Faculty of Medicine, “Iuliu Haţieganu” University of Medicine and Pharmacy, 3-5 Louis Pasteur Street, 400349 Cluj-Napoca, Romania; ioana.ilie@umfcluj.ro

**Keywords:** selective serotonin reuptake inhibitors, drug–drug interactions, antidepressants, pharmacovigilance, real-world evidence

## Abstract

Depression persists as one of the illnesses described relentlessly through the centuries because it affects a large group of people. **Background/Objectives**: The treatment of depression consists of various therapeutic agents, among which selective serotonin reuptake inhibitors (SSRIs) are elective. As polypharmacy tends to become the norm in modern days, the study of the real-life occurrence of drug–drug interactions is imperative. The aim of this study was the evaluation of drug–drug interactions (DDIs) between antidepressant medicines, namely SSRIs (each representative) versus eleven representatives from other antidepressant classes. **Methods**: Based on the spontaneous safety reports (ICSRs) uploaded to EudraVigilance until the end of July 2024, the descriptive and the disproportionality analyses were performed, and results were interpreted in the context of pharmacologic variability. **Results**: SSRIs were the focus of 137,369 ICSRs while for the other antidepressants, namely amitriptyline, clomipramine, duloxetine, venlafaxine, mirtazapine, bupropion, trazodone, tianeptine, agomelatine, brexpiprazole, and esketamine, a total of 155,458 reports were registered. The most notable differences appeared in psychiatric adverse drug reactions. Except fluvoxamine (*n* = 463), the remaining SSRIs had a higher number of DDIs reported (*n* = 1049 for escitalopram and *n* = 1549 for sertraline) compared to other antidepressants. However, similar numbers of DDIs were reported for duloxetine (*n* = 1252) and venlafaxine (*n* = 1513). Sertraline unspecified DDIs were reported with a higher probability compared to all other drugs (e.g., esketamine ROR: 9.37, 95% CI: 5.17–16.96, tianeptine ROR: 4.08, 95% CI: 2.49–6.69, etc.). **Conclusions**: SSRIs, although known to influence various cytochrome P450 isoenzymes, have not shown higher inhibitory interactions compared to any of the drugs selected as reference. Sertraline appears in more reports concerning DDIs than the other antidepressants. Still, further real world studies related to the DDIs of SSRIs are needed to complete the relevant knowledge level.

## 1. Introduction

Depression is a mental illness described since ancient times that negatively influences the quality of life [1]. The hypothesis that depression is caused by an imbalance of serotonin (5-HT) appeared for the first time in 1954, when Freis analyzed the cases of some patients treated for hypertension with reserpine and all patients presented depressive symptoms that disappeared after the drug’s withdrawal. The antihypertensive action of reserpine is modulated by the inhibition of catecholamine reuptake by binding to monoamine transporters [2]. This possible link between depression and cardiovascular diseases is known and has been investigated in several other studies [3,4].

**Figure 1:**
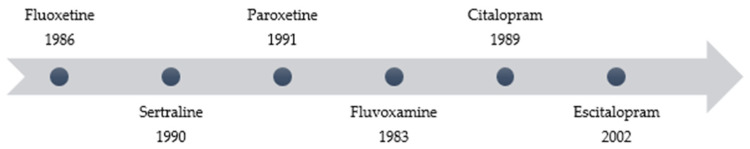
The dynamic of approval dates of SSRI representants [8,9].

For most patients with depression, selective serotonin reuptake inhibitors (SSRIs) are considered first-line pharmacotherapy and are generally appreciated because they are more effective and better tolerated compared to other antidepressants [5]. The mechanism of action of SSRIs is to block the human 5-HT transporter (5-HTT), which increases 5-HT at the synaptic cleft, thereby prolonging the time during which 5-HT activates postsynaptic 5-HT receptors [2]. At the moment, SSRIs represent a group of structurally unrelated molecules, but with a similar mechanism of action, that have been approved for marketing starting with 1997 [6,7] (Figure 1).

Zimelidine was the first SSRI approved in Europe, in 1982. It was synthesized in 1971 by Arvid Carlsson and his colleague, Hans Corrodi, in collaboration with Astra, a Swedish company. Zimelidine, a derivative of pheniramine and inhibitor of the histamine H1 receptor, proved its effectiveness as an antidepressant in clinical studies [2]. Having a pharmacological profile different from that of other antidepressants [10], it stopped being used and was later withdrawn from the market in 1983 after several cases of Guillain–Barré syndrome (SGB), a form of polyneuropathy, were confirmed. Moreover, the cases of SGB were analyzed, and the results showed a risk of developing this syndrome 25 times higher compared to the natural incidence [2,11]. Fluvoxamine was synthetized during the same period as zimelidine, but it was approved in 1983 [8].

In 1974, Wong and his collaborators synthesized another antihistamine with antidepressant properties, fluoxetine, initially calling it Lilly 110140 [2]. It was introduced in therapy in Europe in 1986 [8] and in the United States in 1988 [5]. One of the oldest and best-selling and perhaps the most studied SSRIs, fluoxetine, was initially approved only for premenstrual dysphoric disorder. Currently, other forms of fluoxetine have been approved for use in the treatment of resistant depression, panic disorder, major depressive disorder, etc. With fluoxetine, SSRIs very quickly became the main pharmacotherapy for psychiatric disorders [5] and are prescribed most frequently [12,13] and on a large scale both to patients facing various mental illnesses, such as depression, anxiety, and other mood disorders as well as to patients with emotional disorders characterized by relevant affective symptoms [7,12].

The drug citalopram is the racemic mixture of both the R and S enantiomers of citalopram while escitalopram comprises only the S enantiomer. The S enantiomer is the compound of interest when treating depression while the R enantiomer appears to have no effect and may even interfere with effects of its racemate. Because of its lack of the R enantiomer, escitalopram may be more effective than citalopram for depression and has the highest 5-HTT specificity of the SSRIs [5].

Although these molecules have similar main mechanisms of action, each one has a unique efficacy and pharmacodynamics, pharmacokinetics, and side effect profile, which makes them, to some extent, suitable for a certain clinical niche [5]. SSRIs influence the activity of several cytochrome 450 isoenzymes. Pharmacokinetic and/or pharmacodynamic mechanisms may have clinical implications when antidepressants are administered concomitant to other pharmacological treatments for acute (e.g., COVID-19) or chronic illnesses (e.g., hypercholesterolemia). During the treatment of COVID-19, the drug–drug interactions (DDIs) of SSRIs were mentioned for remdesivir, nirmatrelvir–ritonavir, and, to a lesser extent, for monoclonal antibodies. Severe effects such as serotoninergic syndrome and prolonged QT interval were not excluded. Fluoxetine and paroxetine (strong inhibitors of CYP2D6), and fluvoxamine (a strong inhibitor of CYP1A2 and CYP2C19), were involved with higher frequency in DDIs [14]. Among the new antidepressants, agomelatine, duloxetine, and desvenlafaxine present a lower risk to participate in severe DDIs [15]. The association of antidepressants and several long term treatments such as statins can lead to DDIs, and recommendations are being made on which antidepressants to choose to minimize the risk—escitalopram, mirtazapine, and venlafaxine—or which statins are less subjectable to pharmacokinetic interferences: rosuvastatin or pravastatin [16]. The subject of DDIs for antidepressant medicines is vast due to the rich pharmaceutical arsenal available [17,18].

Despite representing a huge step forward in the management of psychiatric disorders, SSRIs still have a variety of adverse effects that need to be reviewed and monitored [5].

As polypharmacy tends to become the norm in countries with a high standard of living and long-life expectancy, the study of real-life occurrence of DDIs is imperative. The aim of this study was the evaluation of the safety profile segment regarding the drug–drug interactions of antidepressant medicines, namely SSRIs versus representatives from all other antidepressant classes, based on the spontaneous reports registered in EudraVigilance.

## 2. Materials and Methods

### 2.1. Study Design

The Individual Case Safety Reports (ICSRs) submitted to EudraVigilance (EV) pharmacovigilance database for SSRIs were used to evaluate their risk of interactions. Data uploaded to EV portal (https://www.adrreports.eu/, accessed on 1 August 2024) until 28 July 2024 were used for the comparative and disproportionality analysis. All preferred terms (PTs) related to drug–drug interactions (DDIs) were identified. Other PTs, suggesting interactions with disease, food, herbs, alcohol, or tobacco, were excluded. Because ICSRs are anonymous, no ethical approval was required to perform the descriptive or disproportionality analyses.

### 2.2. Material

In EV database, a suspected adverse drug reaction (ADR) is reported under a PT. Also, PT represents a single medical concept and is included in the Medical Dictionary for Regulatory Activities (MedDRA). The related PTs are grouped in the “High Level Terms” group. The highest level of classification is represented by “System Organ Classes” (SOCs) [19]. Each PT is uniquely linked to a single SOC out of 27 SOCs in total. DDIs were grouped into three categories: unspecified drug interaction, inhibitory drug interaction, and potentiating drug interaction. The PTs used to report DDIs are presented in Table 1.

Data reported for the six SSRIs—citalopram, escitalopram, fluoxetine, fluvoxamine, paroxetine, and sertraline—were used in the present study. By comparison with SSRIs, other antidepressant drugs were considered to perform the descriptive and disproportionality analysis: (i) tricyclic antidepressants (amitriptyline and clomipramine), (ii) serotonin/norepinephrine reuptake inhibitors (duloxetine and venlafaxine), (iii) atypical antidepressants (mirtazapine, bupropion and agomelatine), (iv) serotonin modulators (trazodone), (v) N-methyl-D-aspartate receptor antagonists (esketamine), and (vi) others (tianeptine, brexpiprazole) [20].

### 2.3. Descriptive Analysis

First, the study included an analysis of the total number of ICSRs reported for SSRIs. Subsequently, the distribution of reports by seriousness, the number of ADRs reported per case, and the frequency of ADRs reported by SOC were compared between SSRIs and the other antidepressants. In the next step, DDIs reported in EV for antidepressants were evaluated. The comparative analysis was completed by the assessment of the frequency of DDIs in total reported ADRs and the distribution of DDIs by unfavorable outcomes.

### 2.4. Disproportionality Analysis

Disproportionality analysis shows the probability of ADR reporting. As has been stated above, other 11 antidepressant drugs were selected for the disproportionality analysis. In order to perform this analysis using EV data, reporting odds ratio (ROR) and 95% confidence interval (CI) had to be calculated with comparison to all other drugs included in EV or the other drugs from the same therapeutic area or used in the same medical context [21,22,23]. A signal could be considered disproportionate when more than 5 cases were registered for each PT, and lower limit of the 95% CI was higher than 1 [24,25]. MedCalc Software Ltd. (Ostend, Belgium) odds ratio calculator (https://www.medcalc.org/calc/odds_ratio.php (Version 23.1.5; accessed 5 December 2024) [26] was used to calculate the statistical parameters (ROR and 95% CI).

## 3. Results

### Comparative Analysis of Drug–Drug Interactions Reported in EudraVigilance for Selective Serotonin Reuptake Inhibitors and Other Antidepressants

The total number of ICSRs reported for SSRIs and other antidepressant drugs is represented in Supplementary Materials Figure S1. In the SSRI series, the highest number of cases was registered in EV for sertraline (*n* = 35,913), followed by paroxetine (*n* = 28,520), escitalopram (*n* = 24,840), and citalopram (*n* = 22,336). On the other hand, for the other antidepressants, the most numerous cases were reported for venlafaxine (*n* = 36,635), duloxetine (*n* = 33,257), bupropion (*n* = 21,680), and mirtazapine (*n* = 21,466). Also, for agomelatine (*n* = 3588), brexpiprazole (*n* = 2610), esketamine (*n* = 1900), and tianeptine (*n* = 1027) were reported a smaller number of ICSRs than all SSRIs including fluvoxamine (*n* = 4270).

The percentage of cases reported as serious in the SSRI series was 77% (sertraline)–86.9% (fluvoxamine) (Supplementary Materials Figure S2). Although the number of cases for fluvoxamine was the lowest, the percentage of serious cases was the highest. On the other hand, compared to all SSRIs, the percentage of serious cases was higher for brexpiprazole (93.4%), and lower for agomelatine (75.8%), amitriptyline (75.0%), tianeptine (72.5%), and esketamine (59.3%).

According to Supplementary Materials Figure S3, the number of ADRs reported for one case was between 2.3 (sertraline, escitalopram, and citalopram) and 2.6 (paroxetine). For most of the other antidepressants, this number was the same, except for duloxetine (2.7) and agomelatine (2.2), mirtazapine (2.2), and esketamine (2.0).

According to data presented in Supplementary Materials Table S1, some differences regarding the frequency of ADRs reported in different SOCs could be observed between SSRIs and other antidepressants:

- “Cardiac disorders”: higher frequency (amitriptyline–5.5%) and lower frequency (esketamine–1.6% and brexpiprazole–2.1%);
- “Gastrointestinal disorders”: higher frequency (duloxetine–9.7%);
- “General disorders and administration site conditions”: higher frequency (duloxetine–12.8% and venlafaxine–12.8%);
- “Psychiatric disorders”: higher frequency (esketamine–26.9%, tianeptine–17.8%, agomelatine–17.6%, brexpiprazole–17.5%, and trazodone 16.4%) and lower frequency (clomipramine–11.7%).

**Figure 2:**
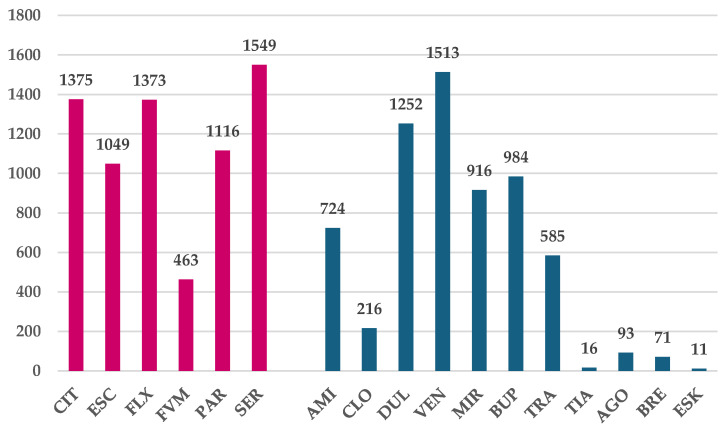
Drug–drug interactions reported in EV for antidepressants. CIT—citalopram, ESC—escitalopram, FXT—fluoxetine, FVM—fluvoxamine, PAR—paroxetine, SER—sertraline, AMI—amitriptyline, CLO—clomipramine, DUL—duloxetine, VEN—venlafaxine, MIR—mirtazapine, BUP—bupropion, TRA—trazodone, TIA—tianeptine, AGO—agomelatine, BRE—brexpiprazole, and ESK—esketamine. Red color—drugs of interest; blue color—drugs used for comparison.

Figure 2 presents the number of DDIs reported in the EV database for SSRIs and other antidepressant drugs. Except fluvoxamine (*n* = 463), SSRIs had a higher number of DDIs reported compared to other antidepressants. However, similar numbers of DDIs were reported for duloxetine (*n* = 1252) and venlafaxine (*n* = 1513).

**Figure 3:**
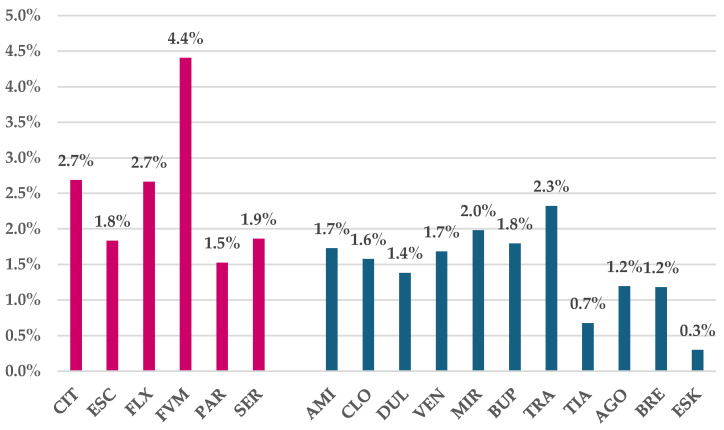
Frequency of DDIs in total ADRs reported in EV database. CIT—citalopram, ESC—escitalopram, FXT—fluoxetine, FVM—fluvoxamine, PAR—paroxetine, SER—sertraline, AMI—amitriptyline, CLO—clomipramine, DUL—duloxetine, VEN—venlafaxine, MIR—mirtazapine, BUP—bupropion, TRA—trazodone, TIA—tianeptine, AGO—agomelatine, BRE—brexpiprazole, and ESK—esketamine. Red color—drugs of interest; blue color—drugs used for comparison.

Regarding the frequency of DDIs in total ADRs, a higher frequency could be noticed for SSRIs by comparison with duloxetine (1.4%), agomelatine and brexpiprazole (1.2%), tianeptine (0.7%), and esketamine (0.3%) (Figure 3).

**Figure 4:**
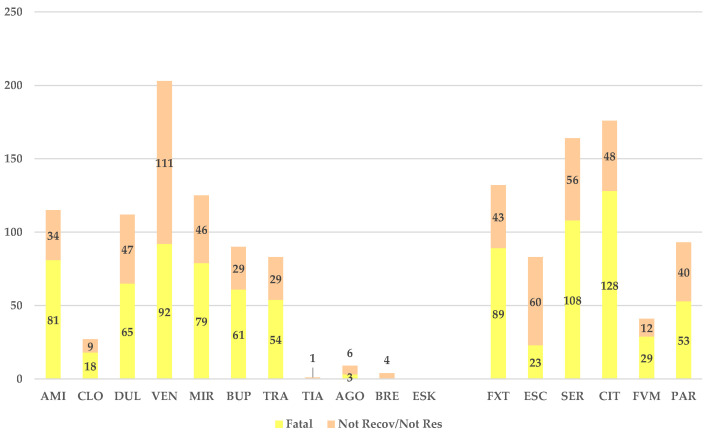
Distribution of ADRs by unfavorable outcomes. CIT—citalopram, ESC—escitalopram, FXT—fluoxetine, FVM—fluvoxamine, PAR—paroxetine, SER—sertraline, AMI—amitriptyline, CLO—clomipramine, DUL—duloxetine, VEN—venlafaxine, MIR—mirtazapine, BUP—bupropion, TRA—trazodone, TIA—tianeptine, AGO—agomelatine, BRE—brexpiprazole, and ESK—esketamine.

A higher number of ADRs with unfavorable outcomes was reported for venlafaxine (*n* = 203) compared to the SSRI series (Figure 4), and for clomipramine (*n* = 27), agomelatine (*n* = 9), brexpiprazole (*n* = 4), tianeptine (*n* = 1), and esketamine (*n* = 0), a lower number of fatal or not-recovered/not-resolved ADRs was reported. Although venlafaxine registered the highest number of ADRs with fatal outcomes, the fatal outcome (*n* = 92) was reported in smaller numbers of cases than for citalopram (*n* = 128) and sertraline (*n* = 108).

**Figure 5:**
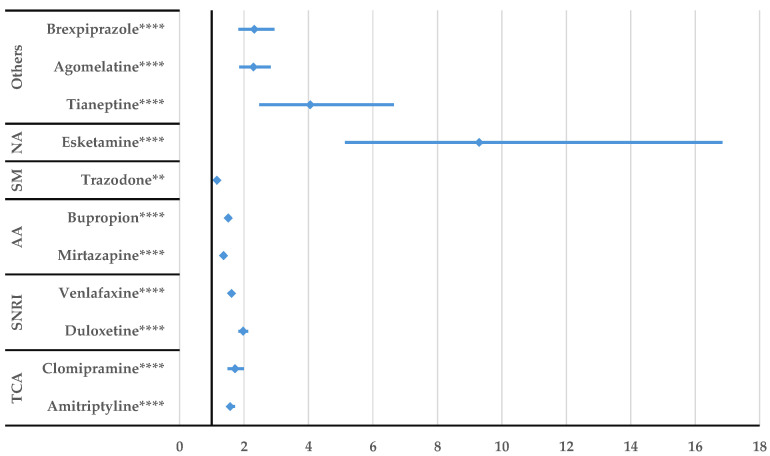
Disproportionality analysis of citalopram unspecified DDIs compared to those of other antidepressants. TCA—tricyclic antidepressants; SNRI—serotonin/norepinephrine reuptake inhibitors; AA—atypical antidepressants; SM—serotonin modulators; NA—N-methyl-D-aspartate receptor antagonists; ** *p* ≤ 0.01; **** *p* ≤ 0.0001.

According to Figure 5, a higher probability of reporting DDIs was observed by comparison with all other comparators. Regarding the potentiating drug interactions, citalopram had a higher probability of reporting compared to other antidepressants such as duloxetine (ROR: 2.07, 95% CI: 1.37–3.15), venlafaxine (ROR: 3.01, 95% CI: 1.89–4.81), and bupropion (ROR: 2.57, 95% CI: 1.53–4.34). Inhibitory citalopram interactions were not reported with a higher probability than for the other drugs (Supplementary Materials Figure S4).

**Figure 6:**
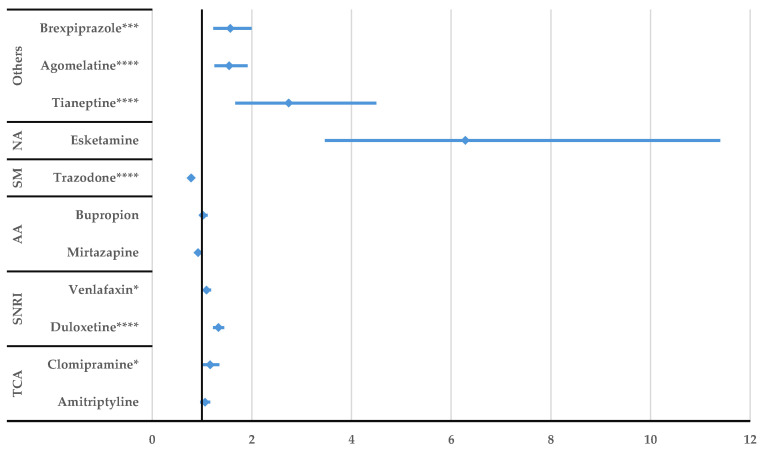
Disproportionality analysis of escitalopram unspecified DDIs compared to those of other antidepressants. TCA—tricyclic antidepressants; SNRI—serotonin/norepinephrine reuptake inhibitors; AA—atypical antidepressants; SM—serotonin modulators; NA—N-methyl-D-aspartate receptor antagonists; * *p* < 0.05; *** *p* ≤ 0.001; **** *p* ≤ 0.0001.

Escitalopram had a lower probability of reporting DDIs compared to trazodone (ROR: 0.78, 95% CI: 0.71–0.87) and higher probability compared to other antidepressants (e.g., duloxetine (ROR: 1.33, 95% CI: 1.23–1.45), venlafaxine (ROR: 1.09; 95% CI: 1.01–1.18), agomelatine (ROR: 1.55, 95% CI: 1.25–1.91), and brexpiprazole (ROR: 1.57, 95% CI: 1.23–1.99) (Figure 6)). Inhibitory or potentiating drug interactions were not reported with a higher probability for escitalopram compared to the other antidepressants (Supplementary Materials Figure S5).

**Figure 7:**
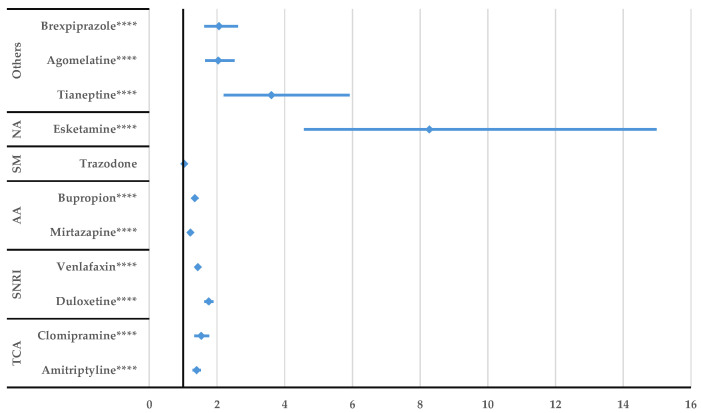
Disproportionality analysis of fluoxetine unspecified DDIs compared to those of other antidepressants. TCA—tricyclic antidepressants; SNRI—serotonin/norepinephrine reuptake inhibitors; AA—atypical antidepressants; SM—serotonin modulators; and NA—N-methyl-D-aspartate receptor antagonists; **** *p* ≤ 0.0001.

Potentiating or inhibitory drug interactions of fluoxetine were not reported more frequently than for the other antidepressants (Supplementary Materials Figure S6), but total DDIs had a higher probability of reporting for fluoxetine compared to all drugs except trazodone (Figure 7).

**Figure 8:**
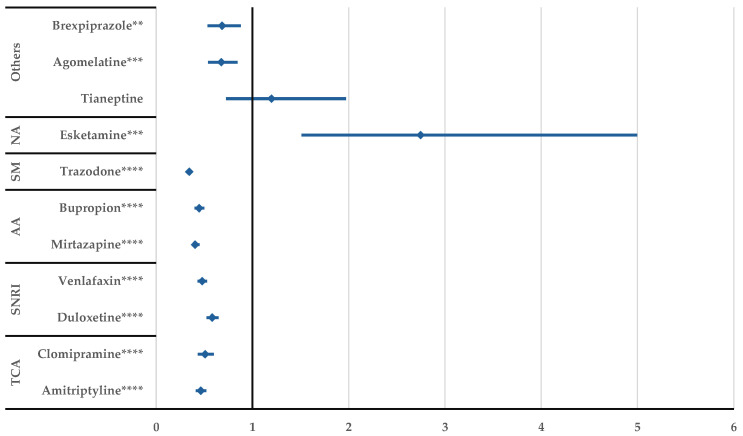
Disproportionality analysis of fluvoxamine unspecified DDIs compared to those of other antidepressants. TCA—tricyclic antidepressants; SNRI—serotonin/norepinephrine reuptake inhibitors; AA—atypical antidepressants; SM—serotonin modulators; NA—N-methyl-D-aspartate receptor antagonists; ** *p* < 0.01; *** *p* ≤ 0.001; **** *p* ≤ 0.0001.

Fluvoxamine had a higher probability of reporting unspecified DDIs only compared to esketamine (ROR: 2.75, 95% CI: 1.51–5.00) (Figure 8). Except esketamine and tianeptine, the DDIs of fluvoxamine had a lower probability of reporting. Also, potentiating fluvoxamine interactions were not reported with a higher probability than those of the other drugs. Moreover, by comparison to trazodone, bupropion, mirtazapine, venlafaxine, and duloxetine, we could observe a lower probability of reporting for fluvoxamine (Supplementary Materials Figure S7).

**Figure 9:**
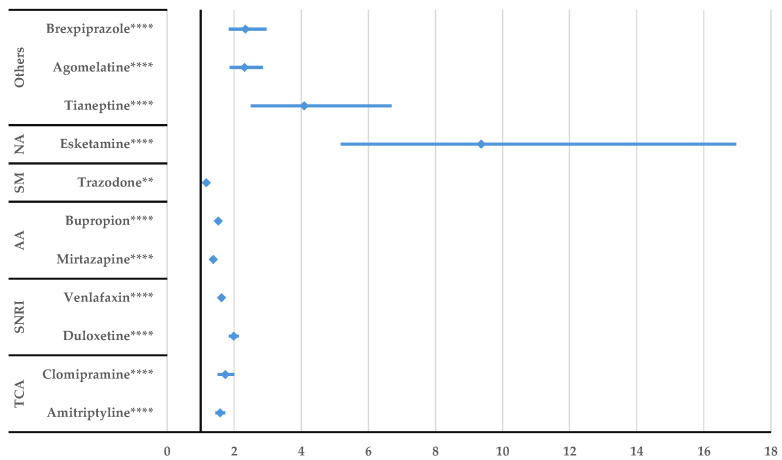
Disproportionality analysis of sertraline unspecified DDIs compared to those of other antidepressants. TCA—tricyclic antidepressants; SNRI—serotonin/norepinephrine reuptake inhibitors; AA—atypical antidepressants; SM—serotonin modulators; NA—N-methyl-D-aspartate receptor antagonists; ** *p* ≤ 0.01; **** *p* ≤ 0.0001.

Potentiating or inhibitory drug interactions of sertraline did not have a higher probability of reporting compared to other drugs (Supplementary Materials Figure S8), but sertraline unspecified DDIs were reported with a higher probability compared to all other drugs (e.g., esketamine ROR: 9.37, 95% CI: 5.17–16.96, tianeptine ROR: 4.08, 95% CI: 2.49–6.69, etc.) (Figure 9).

For paroxetine, no higher probability of reporting inhibitory activity was observed. Comparatively with other antidepressants (e.g., duloxetine (ROR: 0.35, 95% CI: 0.17–0.71), mirtazapine (ROR: 0.25, 95% CI: 0.12–0.53), bupropion (ROR: 0.43, 95% CI: 0.20–0.95), and trazodone (ROR: 0.26, 95% CI: 0.12–0.60)), paroxetine had reported a potentiating activity with higher probability (Supplementary Materials Figure S9).

**Figure 10:**
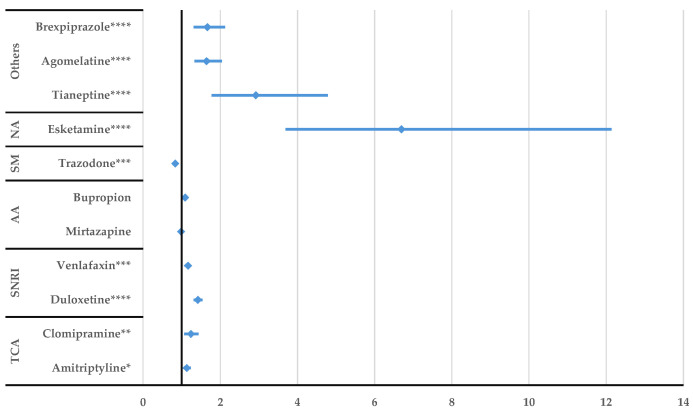
Disproportionality analysis of paroxetine unspecified DDIs compared to those of other antidepressants. TCA—tricyclic antidepressants; SNRI—serotonin/norepinephrine reuptake inhibitors; AA—atypical antidepressants; SM—serotonin modulators; NA—N-methyl-D-aspartate receptor antagonists; * *p* < 0.05; ** *p* ≤ 0.01; *** *p* ≤ 0.001; **** *p* ≤ 0.0001.

The unspecified DDIs of paroxetine had a higher probability of reporting than the majority of antidepressants (e.g., duloxetine (ROR: 1.42, 95% CI: 1.31–1.54), venlafaxine (ROR: 1.16, 95% CI: 1.08–1.26), agomelatine (ROR: 1.65, 95% CI: 1.33–2.04), and esketamine (ROR: 6.70, 95% CI: 3.69–12.14)). On the other hand, compared to trazodone (ROR: 0.84, 95% CI: 0.75–0.92), paroxetine had a higher probability of reporting unspecified DDIs (Figure 10).

## 4. Discussion

At the moment, there are several antidepressants available for therapeutic purposes. Among them, SSRIs are recommended as first-line therapy for depression by several guidelines developed by renowned professional groups [27,28,29]. SSRIs have proven effective for different age groups and ethnicities although disparity has been identified [30,31]. The high number of reports registered for sertraline, paroxetine, and venlafaxine (Figure S1) could be related to the high frequency with which these molecules are prescribed. A recent study measuring the rate of filled prescriptions mentioned all three substances as being among the most prescribed. Four out of the top five antidepressants were SSRIs (sertraline, paroxetine, citalopram, and fluoxetine) and the fifth was venlafaxine [32]. The same molecules seem to have been prescribed and widely accepted by patients for the last five years [33]. For duloxetine, the high number of reports registered confirms the findings of the scientific community. A systematic review of literature concluded that duloxetine was not superior to SSRIs when treating depression and had more side effects [34]. When interpreting the low number of reports registered for agomelatine, brexpiprazole, and esketamine, in comparison to other antidepressants, the time frame of their marketing authorization should also be considered. SSRIs have been available for several decades while agomelatine, brexpiprazole, and esketamine were authorized by the EMA after 2009 [35,36], with esketamine being the newest among the three, having been approved in 2019 [37].

Related to the seriousness of ADRs (Figure S2), some new antidepressants show a better profile in comparison to SSRIs, namely agomelatine (75.8%) and esketamine (59.3%). The situation is reversed for brexpiprazole, where 93.4% of reported ADRs are severe. Recent research on real-world data collected by the Food and Drug Administration Adverse Effects Reporting System (FAERS) has confirmed the occurrence of severe adverse reactions, where brexpiprazole is primarily suspected, alongside several ADRs from the psychiatric and metabolic disorder areas that have not been mentioned by the leaflet [38]. Esketamine has the best ratio of non-serious/serious ADRs, confirming the safety profile described in clinical trials, where mild to moderate AEs were reported the most [39]. By comparison, tianeptine was shown to be well tolerated by several research groups; studies were conducted including different population categories [40,41,42]. Also, agomelatine has a favorable safety profile, a fact supported by the findings of a meta-analysis, where no significant differences could be shown regarding the side effects of agomelatine and those of placebo [43].

The results presented in Figure S3 strengthen the idea of a favorable safety profile of esketamine, a molecule for which the number of ADRs reported by each case was the lowest (2.0). On the opposite side, for duloxetine, the results indicated a higher number of ARs, 2.7 on average. All the SSRIs were situated between the two extremes. When comparing the efficacy and tolerability of several antidepressants, Cipriani et al. also found duloxetine to be less tolerated whereas most SSRIs and agomelatine were accepted better by patients [44].

Noticeable differences have appeared in psychiatric ADRs. Psychiatric unwanted effects seem to be less frequent with SSRIs than with new molecules: esketamine–26.9%, agomelatine–17.6%, and brexpiprazole–17.5%, and even compared to tianeptine–17.8% and trazodone—16.4%. Having been present on the market for a longer period, SSRIs have been studied by various groups of researchers. Regarding the increase of suicidal ideation, one of the most alarming side effects, a recent review expressed that the existing studies, although numerous, could not support a convincing conclusion that linked SSRIs to increased suicidal ideation [5]. For the new antidepressants, our findings support the previously reported data: that for esketamine, agomelatine, and brexpiprazole, psychiatric ADRs are expected [38,45,46].

Some cardiovascular disorders (e.g., orthostatic hypotension, bradycardia, and QT interval prolongation) have been reported under SSRI use [47]. Our study shows that by comparison to SSRIs, the new molecules (e.g., esketamine and brexpiprazole) appear to have a lower frequency of cardiovascular ADRs. On the other hand, for SSRIs, the frequency of ADRs from the “General disorders and administration site conditions” group was lower than for duloxetine and venlafaxine, which could also have had an impact on the reported frequency of DDIs, considering that they were included in this SOC (Table S1). However, the share of DDIs in total ADRs reported for duloxetine and venlafaxine was not higher than for SSRIs; on the contrary, it was lower for duloxetine (1.4%) (Figure 3), suggesting a better safety profile. These findings were in line with the existing data, showing various DDIs being triggered due to SSRIs influencing the activity of several cytochrome P450 isoenzymes and by pharmacodynamic mechanisms [48], while for duloxetine, fewer interactions were described [49], and the same was true for venlafaxine, a molecule considered safer than other antidepressants regarding involvement in clinically significant DDIs [50].

Compared to SSRIs, ADRs with unfavorable outcomes occurred more frequently in amitriptyline (15.9%), trazodone (14.2%), mirtazapine (13.6), and venlafaxine (13.4%), and less often in tianeptine (6.3%), brexpiprazole (5.6%), and esketamine (0%) (Figure 4). The highest frequency of fatal DDIs was reported for amitriptyline (11.2%), citalopram (9.3%), and trazodone (9.2%). For amitriptyline, the scientific literature has shown several cases of fatal DDIs, some of them having occurred in recent years [51,52]. A lower frequency than for SSRIs was recorded for tianeptine, brexpiprazole, and esketamine (0%), and for agomelatine (3.2%), but it was higher only than for escitalopram (2.2%). It seems, therefore, that escitalopram would be the safest regarding fatal DDIs.

No fatal ADRs were reported for tianeptine, brexpiprazole, and esketamine, and for clomipramine (*n* = 38) and agomelatine (*n* = 3), their numbers were lower than for any of the SSRIs. Thus, brexpiprazole and agomelatine seem to be safer than SSRIs when considering the outcome of the ADRs.

After performing the disproportionality analysis comparing each DDI of the SSRIs to other representative antidepressants (Figure 5, Figure 6, Figure 7, Figure 8, Figure 9 and Figure 10), several situations were noted. Unspecified DDIs appeared more frequently reported for citalopram than for the antidepressants used for comparison. The present data showed that citalopram had a more frequently reported potentiation activity than amitriptyline, duloxetine, venlafaxine, and bupropion. The identification of possible interactions between antidepressants and several therapeutical classes is a subject of interest for the scientific community, with methods being developed to identify these shortcomings from the preclinical stage [53]. For escitalopram, a higher probability of reporting unspecified DDIs was found compared to clomipramine, duloxetine, venlafaxine, tianeptine, agomelatine, and brexpiprazole, but escitalopram had a lower probability of reporting DDIs than trazodone. Also, fluoxetine presented DDIs more frequently than all drugs except trazodone. This outcome could be linked to the high prescription rate of this drug to patients with comorbidities and the pharmacokinetic profile of fluoxetine as described by Malavika et al. [54]. On the other hand, fluvoxamine had a lower probability of reporting compared to the other antidepressants, except for esketamine and tianeptine. Also, compared to trazodone, bupropion, mirtazapine, venlafaxine, and duloxetine, potentiation interactions were less frequently reported for fluvoxamine.

DDIs were more frequently reported for sertraline than any other antidepressant. The pharmacokinetics of sertraline involve several isoenzymes, among which CYP2D6, CYP2C19, and CYP2B6, the major metabolic pathway being CYP2C19, are involved in the metabolization of other drugs and can lead to clinical significant interactions [55]. Paroxetine showed potentiation interaction more frequently than duloxetine, mirtazapine, bupropion, and trazodone. Trazodone has a higher reporting probability than paroxetine for DDIs. Paroxetine is more likely to report DDIs than most antidepressants used for comparison: amitriptyline, clomipramine, duloxetine, venlafaxine, tianeptine, agomelatine, brexpiprazole, and esketamine.

Also, the present results showed that no SSRI has inhibitory interactions compared to any of the drugs selected as reference. In order to prevent interactions, the different genotypes of the enzymes involved in the metabolization of antidepressants should be investigated and therapeutical doses adjusted accordingly [56].

One of the main concerns for clinicians when prescribing SSRIs is their potential for drug–drug interactions [57]. Other classes of antidepressants, some of them newly authorized, with diverse mechanisms of action, are available. A real-world evidence comparison regarding drug–drug interaction prospects is beneficial. It offers further support for clinical decisions in selecting a safer alternative when needed.

The fact that several research groups have analyzed spontaneous reports shows the high analytic potential these regularly updated and curated databases carry. New characteristics of the examined subject are exposed by differentiating the comparison items and the statistical tools.

The limitations of the study derived from the data accessible on EudraVigilance, where information regarding the number of patients treated with antidepressants in the studied period is not available [58]. Other information that could not be retrieved included the following: the ages of the patients, ethnicities, diagnoses, existing comorbidities, and other medication administered. Thus, a direct causal relation cannot, and is recommended by EV itself not to, be considered. Other serious limitations that may have weakened the validity of the obtained conclusions were the inability to take into account the time frames of use of the products, unknown doses and lengths of therapy in patients, etc. After taking into account these parameters, it would be possible to establish real indicators of the products used.

## 5. Conclusions

Depression is a widespread medical condition. SSRIs are considered first-line therapy. Because polymedication is often present in modern-day patients, this study aimed to evaluate the real-world evidence of drug–drug interactions related to SSRIs and other antidepressants based on the spontaneous reports available in EudraVigilance. ADRs were registered for all the substances of interest for this study. SSRIs, although known to influence various cytochrome P450 isoenzymes, have not shown higher inhibitory interactions compared to any of the drugs selected as reference. Sertraline appears in more reports concerning DDIs than the other antidepressants. Similar, fluoxetine presents unspecified DDIs more frequently than all drugs except trazodone. Still, further real world studies related to the DDIs of SSRIs are needed to complete the relevant knowledge level.

## Figures and Tables

**Table 1:** Preferred terms related to DDIs.

Category of DDIs	PTs
Unspecified drug interaction	Drug interaction Labelled drug–drug interaction issue Labelled drug–drug interaction medication error
Inhibitory drug interaction	Inhibitory drug interaction
Potentiating drug interaction	Potentiating drug interaction

## Data Availability

Data are contained within the article.

## Supplementary Materials

The following supporting information can be downloaded at: https://www.mdpi.com/article/10.3390/jcm14041208/s1, Figure S1: Total number of ICSRs reported for SSRIs compared to other antidepressants; Figure S2: Distribution by the seriousness of ICSRs reported for SSRIs compared to other antidepressants; Figure S3: Number of ADRs reported per one case; Figure S4: Disproportionality analysis of citalopram DDI compared to other antidepressants; Figure S5: Disproportionality analysis of escitalopram DDI compared to other antidepressants; Figure S6: Disproportionality analysis of fluoxetine DDI compared to other antidepressants; Figure S7: Disproportionality analysis of fluvoxamine potentianing DDI compared to other antidepressants; Figure S8: Disproportionality analysis of fluvoxamine DDI compared to other antidepressants; Figure S9: Disproportionality analysis of fluvoxamine DDI compared to other antidepressants; Table S1: The frequency of ADRs reported by System Organ Classes (SOC).

## Abbreviations

The following abbreviations have been used in this manuscript.

5-HT	serotonin
5-HTT	5-HT transporter
ADRs	adverse drug reactions
AGO	agomelatine
AMI	amitriptyline
BRE	brexpiprazole
BUP	bupropion
CI	confidence interval
CIT	citalopram
CLO	clomipramine
DDI	drug–drug interactions
DUL	duloxetine
ESC	escitalopram
ESK	esketamine
EV	EudraVigilance
FVM	fluvoxamine
FXT	fluoxetine
ICSRs	individual case safety reports
MedDRA	Medical Dictionary for Regulatory Activities
MIR	mirtazapine
NS	not specified
PAR	paroxetine
PT	preferred term
ROR	reporting odds ratio
SER	sertraline
SOC	system organ class
SSRIs	selective serotonin reuptake inhibitors
TIA	tianeptine
TRA	trazadone
VEN	venlafaxine
