# Supplementary material for: The Assessment of the Safety Profile of Selective Serotonin Reuptake Inhibitors Versus Other Antidepressants: Drug–Drug Interaction Insights from EudraVigilance
Source: J Clin Med. 2025 Feb 12;14(4):1208. doi: 10.3390/jcm14041208 (PMC11856802; doi:10.3390/jcm14041208)
Supplement: Supplementary file 1 [file jcm-14-01208-s001.zip › jcm-3449339-supplementary.pdf]

SUPPLEMENTARY MATERIALS

The Assessment of the Safety Profile of Selective Serotonin Reuptake Inhibitors versus Other Antidepressants: Drug-Drug Interactions Insights from EudraVigilance

Carmen Maximiliana Dobrea [1,†], Claudiu Morgovan [1,†], Adina Frum [1,*], Anca Butuca [1,*], Adriana Aurelia Chis [1], Anca Maria Arseniu [1], Steliana Ghibu [2], Razvan Constantin Vonica [1], Felicia Gabriela Gligor [1], Ioana Rada Popa Ilie [3], Andreea Loredana Vonica-Tincu [1]

1 Preclinical Department, Faculty of Medicine, “Lucian Blaga” University of Sibiu, 550169 Sibiu, Romania;
2 Department of Pharmacology, Physiology and Pathophysiology, Faculty of Pharmacy, “Iuliu Hatieganu” University of Medicine and Pharmacy, 400012 Cluj-Napoca, Romania;
3 Department of Endocrinology, Faculty of Medicine, “Iuliu Haţieganu” University of Medicine and Pharmacy, 3-5 Louis Pasteur Street, 400349 Cluj-Napoca, Romania;
* Correspondence: adina.frum@ulbsibiu.ro (A.F.); anca.butuca@ulbsibiu.ro (A.B.);
† These authors contributed equally to this work.

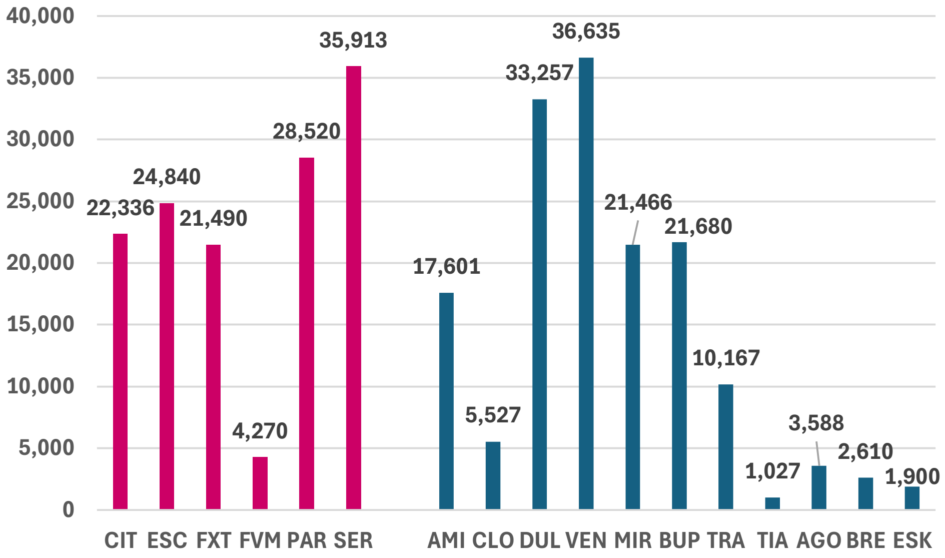

Figure S1. Total number of ICSRs reported for SSRIs compared to other antidepressants. CIT -citalopram, ESC – escitalopram, FXT – fluoxetine, FVM - fluvoxamine, PAR – paroxetine, SER – sertraline, AMI - amitriptyline, CLO - clomipramine, DUL - duloxetine, VEN - venlafaxine, MIR -mirtazapine, BUP - bupropion, TRA - trazodone, TIA – tianeptine, AGO – agomelatine, BRE - brexpiprazole, ESK – esketamine.

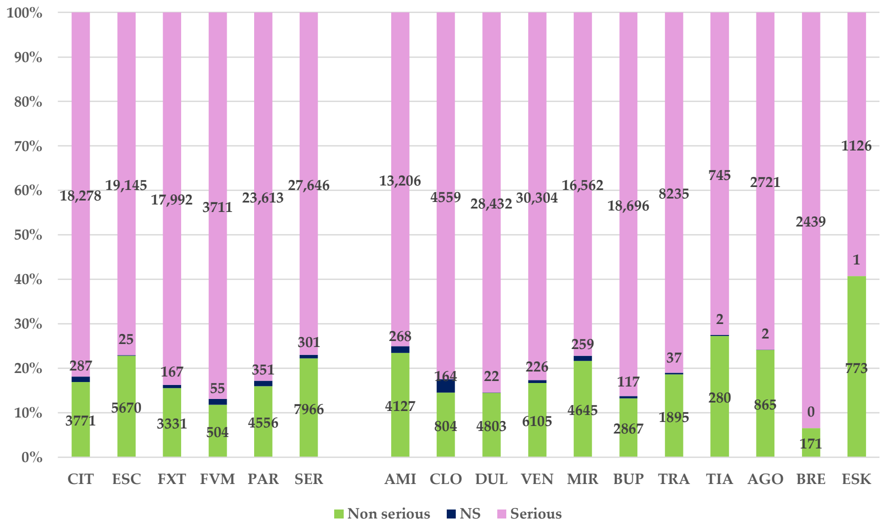

Figure S2. Distribution by the seriousness of ICSRs reported for SSRIs compared to other antidepressants. CIT - citalopram, ESC – escitalopram, FXT – fluoxetine, FVM - fluvoxamine, PAR – paroxetine, SER – sertraline, AMI - amitriptyline, CLO - clomipramine, DUL - duloxetine, VEN - venlafaxine, MIR -mirtazapine, BUP - bupropion, TRA - trazodone, TIA – tianeptine, AGO – agomelatine, BRE - brexpiprazole, ESK – esketamine; NS – not specified.

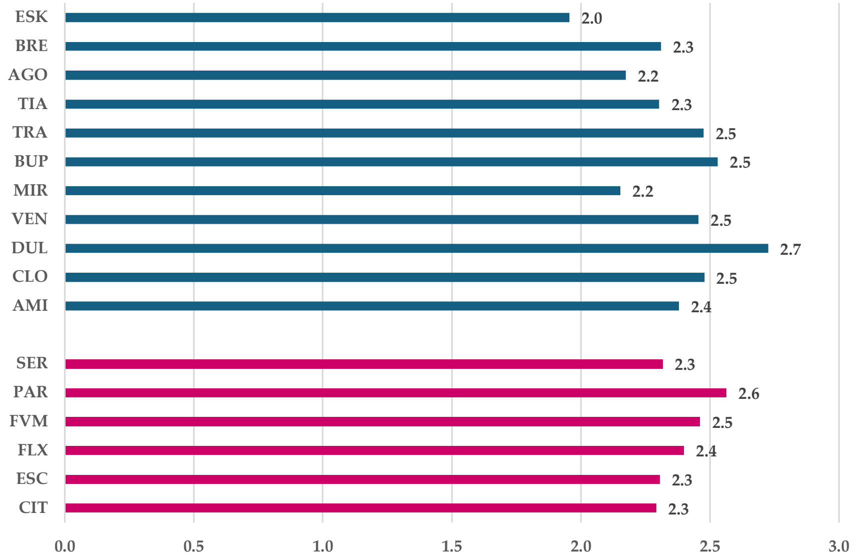

Figure S3. Number of ADRs reported per one case. CIT -citalopram, ESC – escitalopram, FXT – fluoxetine, FVM - fluvoxamine, PAR – paroxetine, SER – sertraline, AMI - amitriptyline, CLO - clomipramine, DUL - duloxetine, VEN - venlafaxine, MIR -mirtazapine, BUP - bupropion, TRA - trazodone, TIA – tianeptine, AGO – agomelatine, BRE - brexpiprazole, ESK – esketamine.

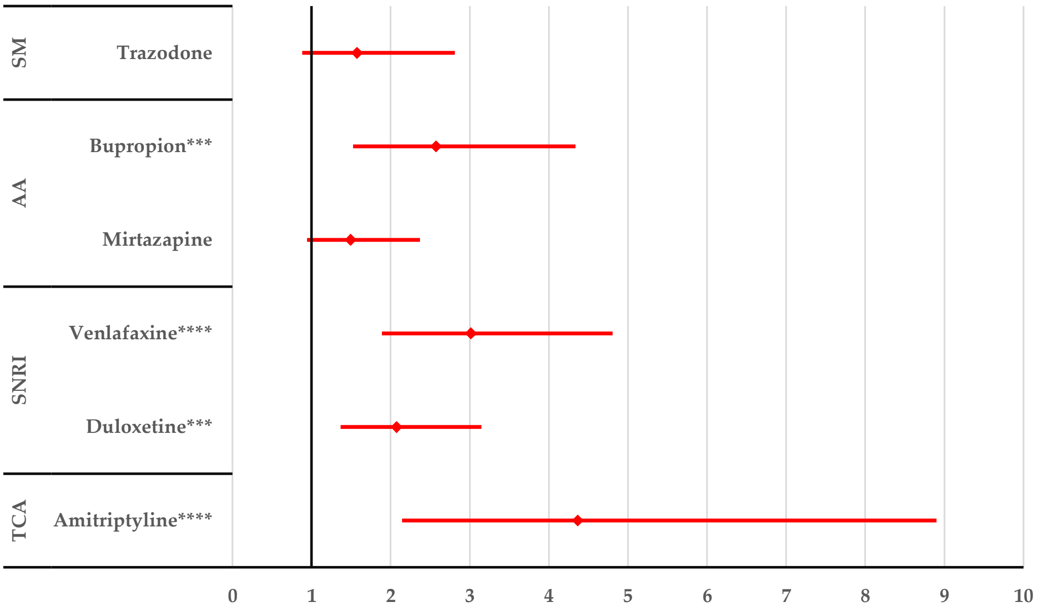

(a)

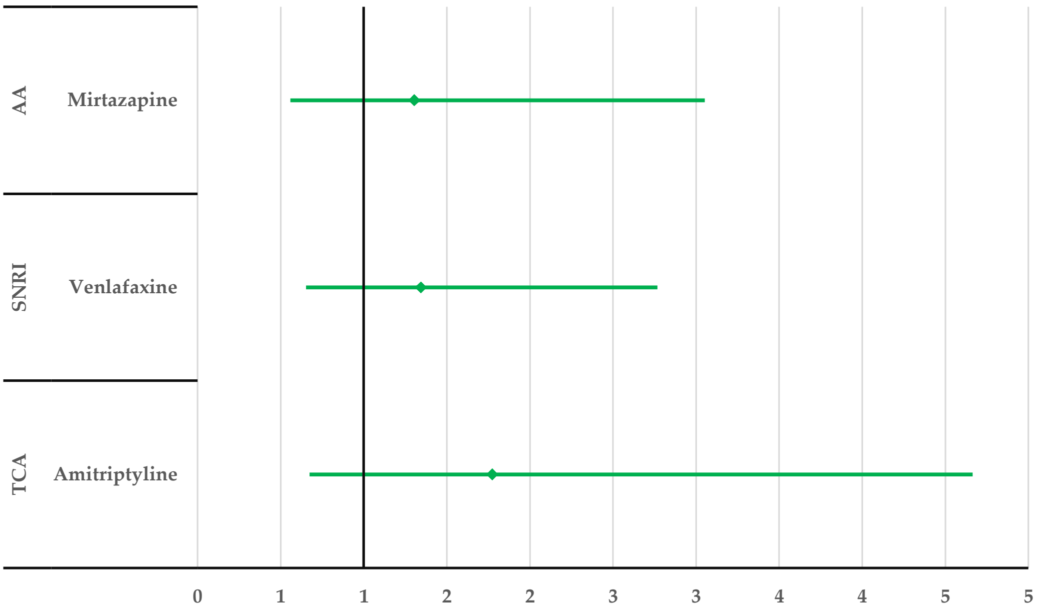

(b)

Figure S4. Disproportionality analysis of citalopram DDI compared to other antidepressants. (a) – potentiating drug interaction; (b) - inhibitory drug interaction; TCA - tricyclic antidepressants; SNRI - serotonin/norepinephrine reuptake inhibitors; AA - atypical antidepressants; SM - serotonin modulators; NA - N-methyl-D-aspartate receptor antagonists; *** $p \leq 0.001$; **** $p \leq 0.0001$.

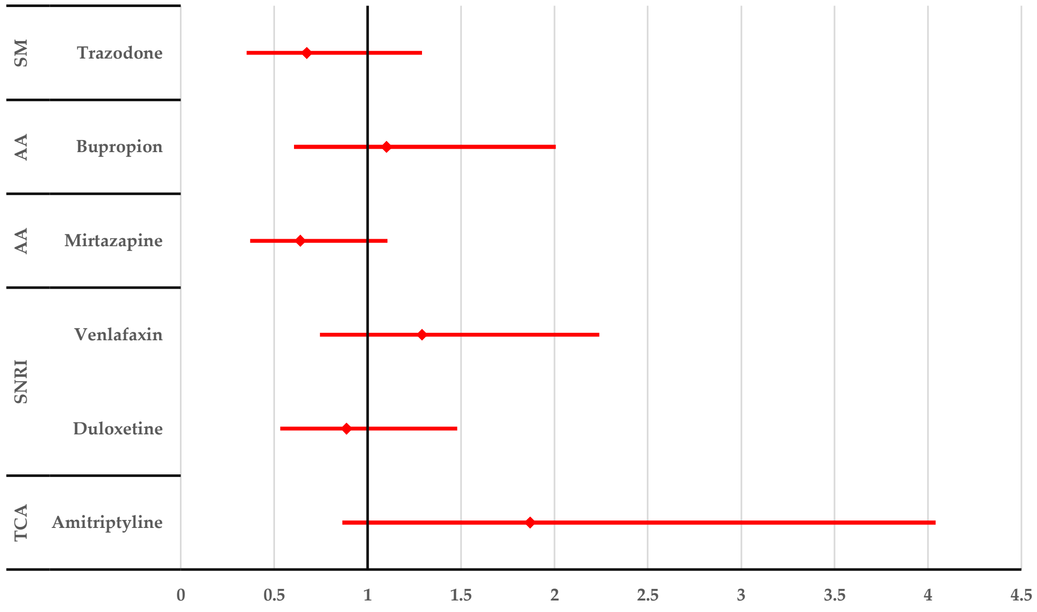

(a)

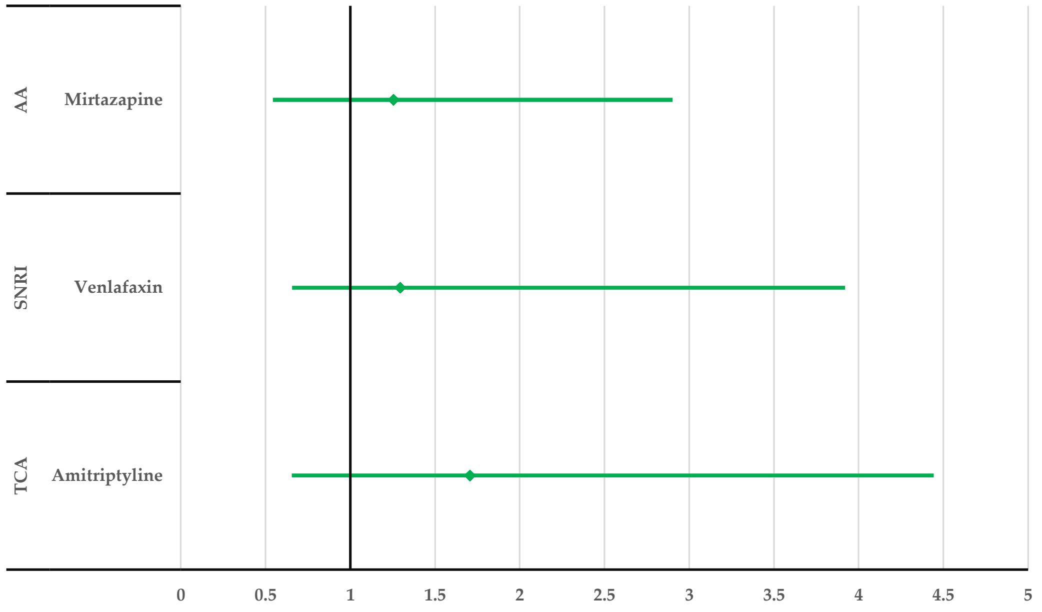

(b)

Figure S5. Disproportionality analysis of escitalopram DDI compared to other antidepressants. (a) – potentiating drug interaction; (b) - inhibitory drug interaction; TCA - tricyclic antidepressants; SNRI - serotonin/norepinephrine reuptake inhibitors; AA - atypical antidepressants; SM - serotonin modulators; NA - N-methyl-D-aspartate receptor antagonists.

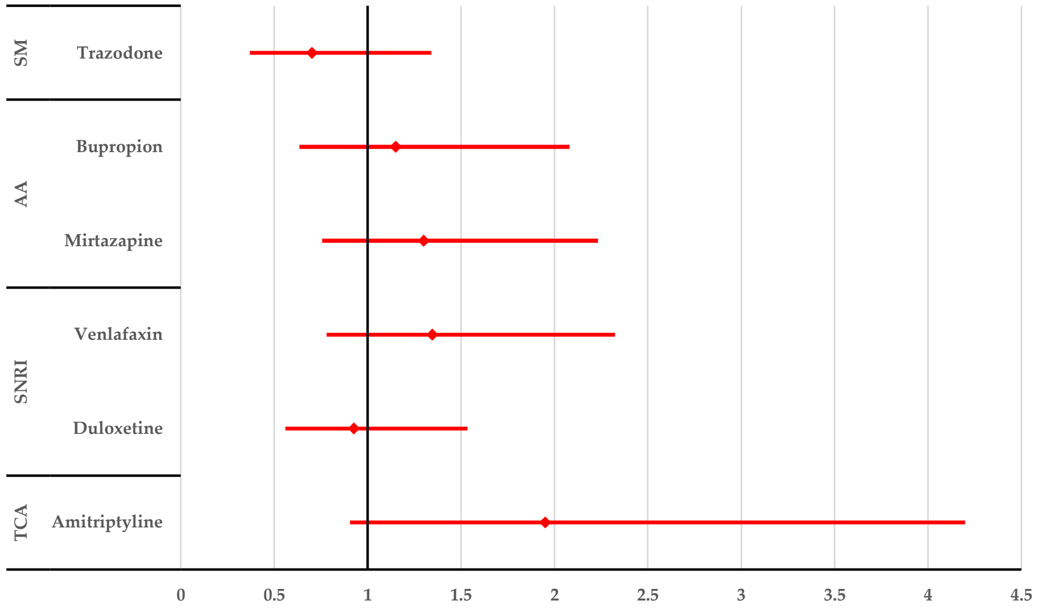

(a)

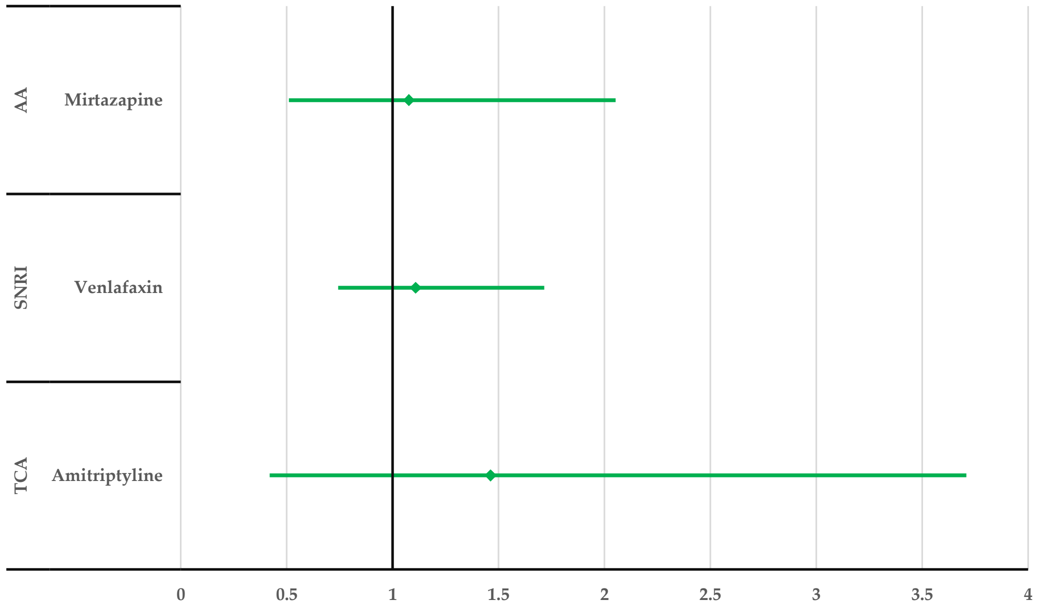

(b)

Figure S6. Disproportionality analysis of fluoxetine DDI compared to other antidepressants. (a) – potentiating drug interaction; (b) - inhibitory drug interaction; TCA - tricyclic antidepressants; SNRI - serotonin/norepinephrine reuptake inhibitors; AA - atypical antidepressants; SM - serotonin modulators; NA - N-methyl-D-aspartate receptor antagonists.

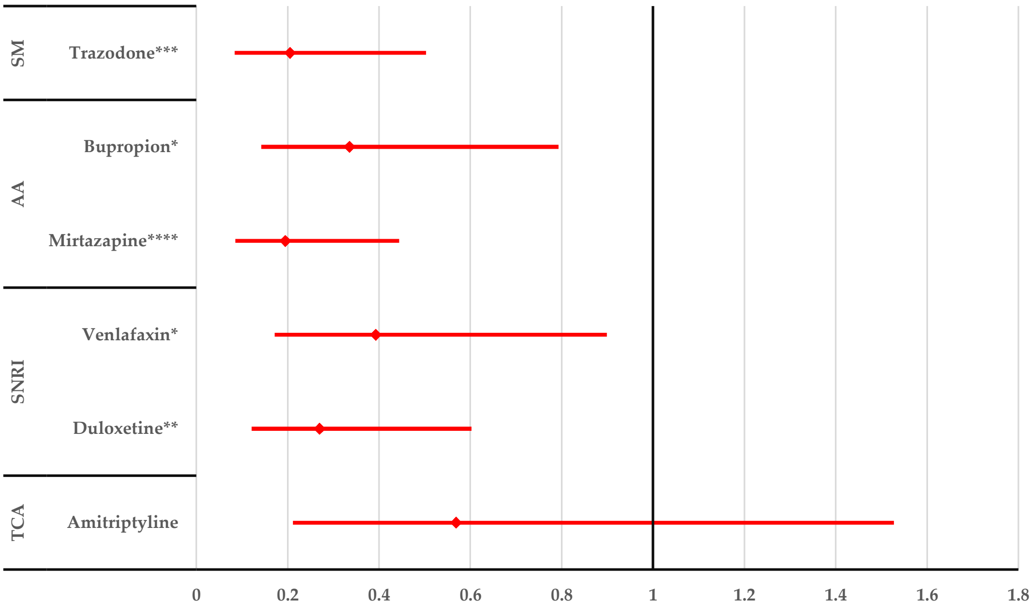

Figure S7. Disproportionality analysis of fluvoxamine potentianing DDI compared to other antidepressants. TCA - tricyclic antidepressants; SNRI - serotonin/norepinephrine reuptake inhibitors; AA - atypical antidepressants; SM - serotonin modulators; NA - N-methyl-D-aspartate receptor antagonists.

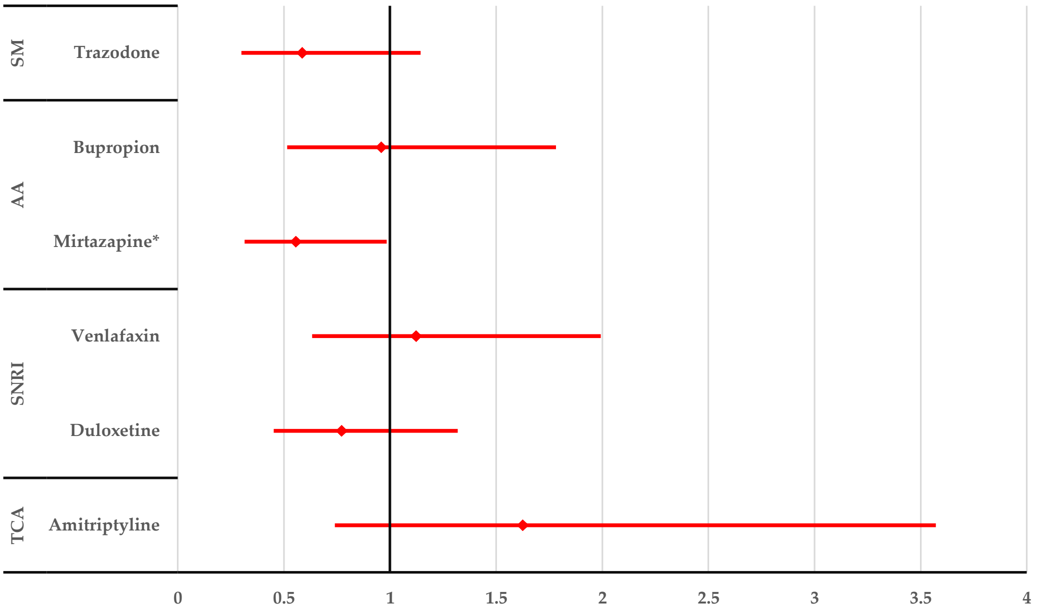

(a)

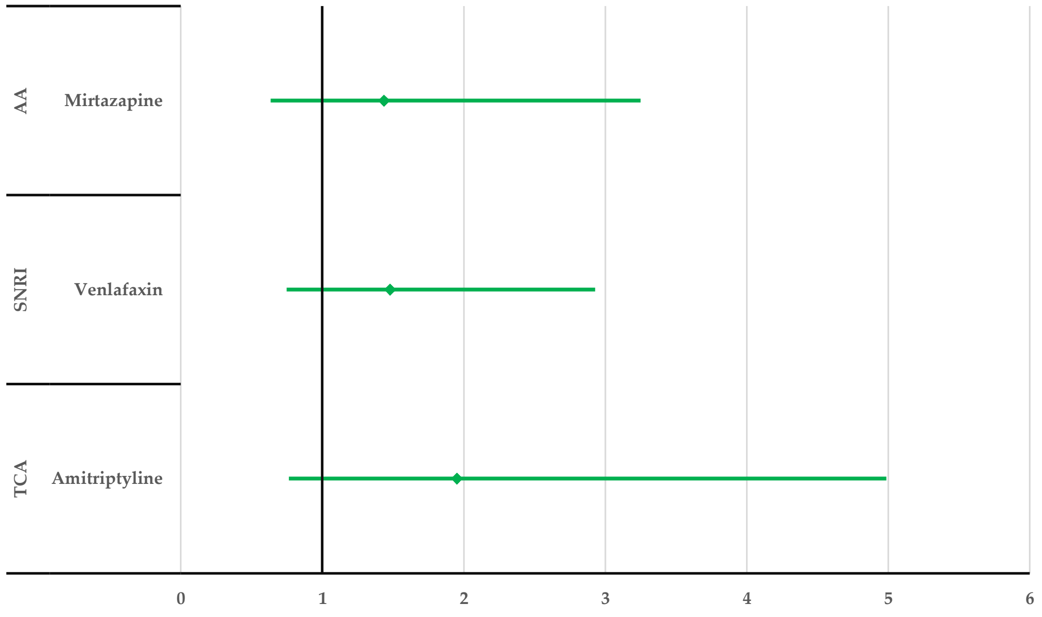

(b)

Figure S8. Disproportionality analysis of fluvoxamine DDI compared to other antidepressants. (a) – potentiating drug interaction; (b) - inhibitory drug interaction; TCA - tricyclic antidepressants; SNRI - serotonin/norepinephrine reuptake inhibitors; AA - atypical antidepressants; SM - serotonin modulators; NA - N-methyl-D-aspartate receptor antagonists. * $p < 0.05$.

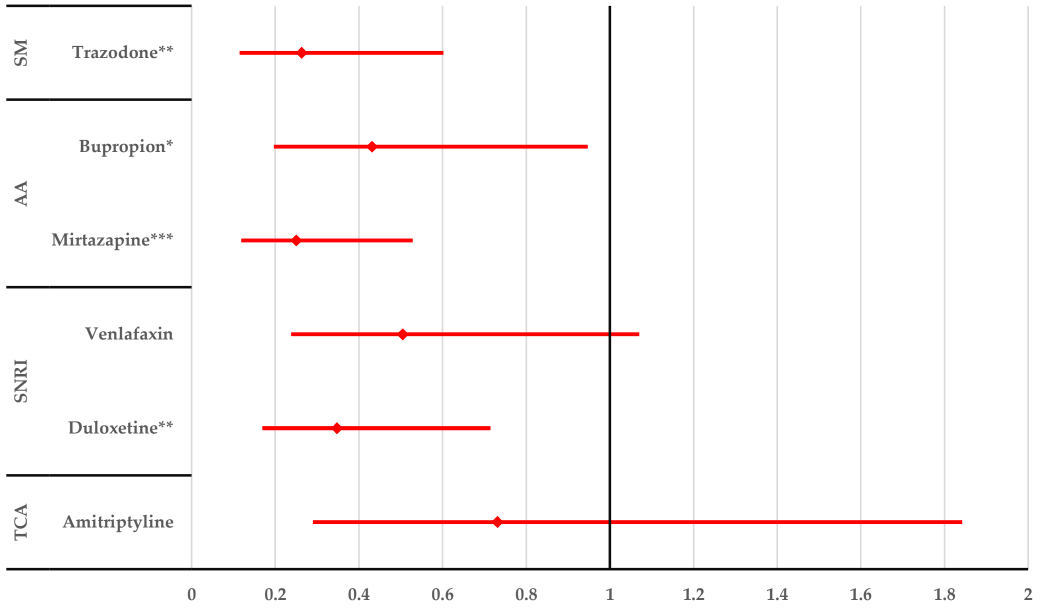

(a)

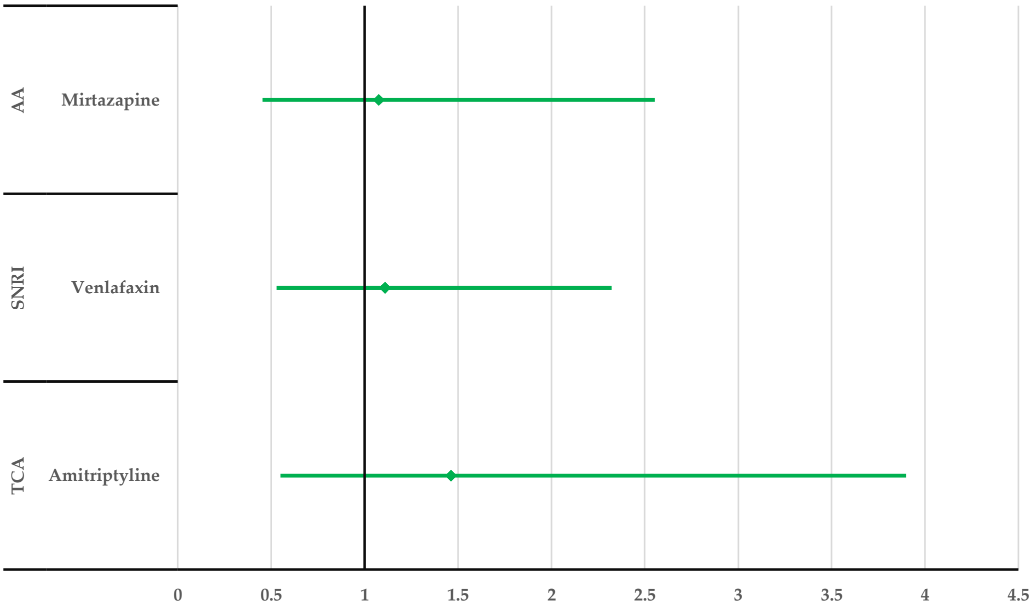

(b)

Figure S9. Disproportionality analysis of fluvoxamine DDI compared to other antidepressants. (a) – potentiating drug interaction; (b) - inhibitory drug interaction; TCA - tricyclic antidepressants; SNRI - serotonin/norepinephrine reuptake inhibitors; AA - atypical antidepressants; SM - serotonin modulators; NA - N-methyl-D-aspartate receptor antagonists. * $p < 0.05$; ** $p \leq 0.01$; *** $p \leq 0.001$.

Table S1. The frequency of ADRs reported by System Organ Classes (SOC). CIT -citalopram, ESC – escitalopram, FXT – fluoxetine, FVM - fluvoxamine, PAR – paroxetine, SER – sertraline, AMI - amitriptyline, CLO - clomipramine, DUL - duloxetine, VEN - venlafaxine, MIR -mirtazapine, BUP - bupropion, TRA - trazodone, TIA – tianeptine, AGO – agomelatine, BRE - brexpiprazole, ESK – esketamine.

SOC	CIT	ESC	FLX	FVM	PAR	SER	AMI	CLO	DUL	VEN	MIR	BUP	TRA	TIA	AGO	BRE	ESK
**Blood and lymphatic system disorders**	1.5%	1.3%	1.4%	1.8%	1.2%	1.1%	1.1%	2.5%	0.6%	1.1%	2.2%	0.9%	1.1%	1.6%	1.0%	0.7%	0.2%
**Cardiac disorders**	5.0%	3.8%	4.3%	3.8%	4.0%	3.5%	5.5%	4.6%	2.7%	4.2%	3.6%	4.6%	5.0%	3.6%	2.6%	2.1%	1.6%
**Congenital, familial and genetic disorders**	1.0%	1.1%	2.9%	0.4%	5.5%	2.0%	0.4%	0.8%	0.2%	0.7%	0.3%	0.3%	0.2%	0.2%	0.2%	0.1%	0.1%
**Ear and labyrinth disorders**	0.8%	1.1%	0.6%	0.5%	1.2%	1.1%	1.0%	0.7%	3.3%	1.2%	0.8%	1.1%	0.7%	1.4%	1.2%	0.2%	1.4%
**Endocrine disorders**	0.6%	0.8%	0.6%	0.7%	0.8%	0.8%	0.4%	0.6%	0.9%	0.5%	0.6%	0.3%	0.5%	0.5%	0.3%	0.5%	0.1%
**Eye disorders**	1.7%	1.8%	1.7%	1.6%	1.9%	2.1%	2.1%	2.0%	2.2%	2.3%	1.7%	1.8%	1.7%	2.0%	1.7%	1.9%	2.4%
**Gastrointestinal disorders**	6.5%	7.5%	5.6%	6.3%	6.3%	8.8%	6.5%	5.4%	9.7%	6.7%	5.9%	6.3%	5.9%	7.3%	7.8%	4.2%	6.9%
**General disorders and administration site conditions**	11.7%	11.4%	11.2%	12.5%	10.9%	11.5%	12.0%	11.5%	12.8%	12.8%	12.2%	11.1%	11.8%	11.2%	10.5%	12.4%	12.6%
**Hepatobiliary disorders**	0.8%	1.0%	1.1%	1.9%	1.3%	1.2%	1.2%	2.0%	1.3%	1.1%	1.4%	0.6%	1.2%	1.9%	3.8%	0.6%	0.6%
**Immune system disorders**	0.6%	0.6%	0.7%	0.3%	0.3%	0.6%	0.6%	0.3%	0.7%	0.7%	0.4%	1.8%	0.8%	0.2%	0.3%	0.7%	0.8%
**Infections and infestations**	1.4%	1.3%	1.5%	1.5%	1.4%	1.6%	1.6%	1.2%	1.7%	1.6%	1.6%	1.7%	1.6%	1.2%	1.6%	1.6%	1.5%
**Injury, poisoning and**	10.6%	9.2%	12.3%	8.4%	11.6%	9.1%	12.7%	8.8%	7.6%	9.4%	9.9%	9.6%	11.7%	10.2%	8.0%	13.7%	6.6%

SOC	CIT	ESC	FLX	FVM	PAR	SER	AMI	CLO	DUL	VEN	MIR	BUP	TRA	TIA	AGO	BRE	ESK
**procedural complications**																	
**Investigations**	5.7%	5.6%	5.5%	8.9%	5.3%	5.0%	5.3%	7.7%	5.3%	5.9%	6.5%	5.2%	5.1%	5.5%	12.0%	7.1%	5.8%
**Metabolism and nutrition disorders**	4.4%	4.2%	3.4%	3.4%	3.6%	3.5%	2.3%	3.7%	3.2%	3.2%	4.0%	4.6%	2.6%	4.7%	2.4%	2.6%	0.8%
**Musculoskeletal and connective tissue disorders**	2.8%	3.1%	2.8%	2.7%	2.9%	3.4%	2.9%	2.9%	3.2%	3.2%	3.6%	3.4%	3.2%	2.5%	3.2%	2.9%	1.3%
**Neoplasms benign, malignant and unspecified (incl cysts and polyps)**	0.3%	0.3%	0.5%	0.2%	0.3%	0.6%	0.4%	0.7%	0.6%	0.5%	0.3%	0.5%	0.3%	0.4%	0.7%	0.6%	0.2%
**Nervous system disorders**	13.5%	14.5%	12.4%	17.4%	13.4%	13.8%	15.1%	15.8%	15.3%	14.3%	16.2%	14.2%	14.4%	14.3%	12.9%	18.7%	17.8%
**Pregnancy, puerperium and perinatal conditions**	0.9%	0.9%	1.9%	0.4%	1.1%	1.0%	0.4%	1.2%	0.5%	0.9%	0.6%	0.6%	0.6%	0.4%	0.4%	0.3%	0.1%
**Product issues**	0.3%	0.6%	0.5%	0.2%	0.5%	0.6%	0.4%	0.4%	0.3%	0.5%	0.5%	0.5%	0.4%	0.2%	0.3%	0.3%	0.8%
**Psychiatric disorders**	15.9%	15.4%	15.5%	14.5%	13.3%	14.5%	13.5%	11.7%	14.0%	14.5%	15.4%	14.6%	16.4%	17.8%	17.6%	17.5%	26.9%
**Renal and urinary disorders**	1.2%	1.7%	1.4%	2.0%	1.5%	1.5%	1.9%	2.2%	1.9%	1.9%	1.8%	2.2%	1.7%	1.3%	1.4%	1.0%	1.2%
**Reproductive system and breast disorders**	1.7%	2.1%	1.4%	1.0%	1.3%	2.0%	0.7%	1.0%	0.8%	1.2%	0.8%	0.8%	2.0%	0.5%	0.9%	0.9%	0.2%
**Respiratory, thoracic and mediastinal disorders**	3.5%	2.9%	3.7%	2.9%	3.4%	3.5%	4.2%	3.7%	2.5%	3.3%	3.1%	3.7%	4.1%	2.8%	1.4%	1.5%	3.4%

SOC	CIT	ESC	FLX	FVM	PAR	SER	AMI	CLO	DUL	VEN	MIR	BUP	TRA	TIA	AGO	BRE	ESK
**Skin and subcutaneous tissue disorders**	3.8%	4.2%	3.5%	3.3%	3.5%	4.2%	3.6%	4.1%	5.2%	4.2%	3.4%	5.6%	2.9%	4.9%	4.4%	1.7%	2.2%
**Social circumstances**	0.5%	0.7%	0.5%	0.2%	0.7%	0.6%	0.6%	0.2%	0.4%	0.7%	0.4%	0.7%	0.8%	0.6%	1.0%	2.2%	0.4%
**Surgical and medical procedures**	0.5%	0.6%	0.5%	0.3%	0.5%	0.4%	0.4%	0.6%	0.8%	0.4%	0.4%	0.6%	0.5%	0.1%	0.6%	2.4%	0.9%
**Vascular disorders**	2.6%	2.4%	2.3%	2.6%	2.2%	2.1%	3.1%	4.0%	2.5%	3.0%	2.4%	2.7%	2.8%	2.7%	1.9%	1.5%	3.5%
